# Genome-Wide Transcriptional Analysis Reveals the Protection against Hypoxia-Induced Oxidative Injury in the Intestine of Tibetans via the Inhibition of GRB2/EGFR/PTPN11 Pathways

**DOI:** 10.1155/2016/6967396

**Published:** 2016-08-09

**Authors:** Kang Li, Luobu Gesang, Zeng Dan, Lamu Gusang

**Affiliations:** ^1^High Altitude Medical Research Institute, People's Hospital of Tibet Autonomous Region, Lhasa 850000, China; ^2^Department of Cardiology, People's Hospital of Tibet Autonomous Region, Lhasa 850000, China; ^3^Department of Gastroenterology, People's Hospital of Tibet Autonomous Region, Lhasa 850000, China

## Abstract

The molecular mechanisms for hypoxic environment causing the injury of intestinal mucosal barrier (IMB) are widely unknown. To address the issue, Han Chinese from 100 m altitude and Tibetans from high altitude (more than 3650 m) were recruited. Histological and transcriptome analyses were performed. The results showed intestinal villi were reduced and appeared irregular, and glandular epithelium was destroyed in the IMB of Tibetans when compared with Han Chinese. Transcriptome analysis revealed 2573 genes with altered expression. The levels of 1137 genes increased and 1436 genes decreased in Tibetans when compared with Han Chinese. Gene ontology (GO) analysis indicated most immunological responses were reduced in the IMB of Tibetans when compared with Han Chinese. Gene microarray showed that there were 25-, 22-, and 18-fold downregulation for growth factor receptor-bound protein 2 (GRB2), epidermal growth factor receptor (EGFR), and tyrosine-protein phosphatase nonreceptor type 11 (PTPN11) in the IMB of Tibetans when compared with Han Chinese. The downregulation of EGFR, GRB2, and PTPN11 will reduce the production of reactive oxygen species and protect against oxidative stress-induced injury for intestine. Thus, the transcriptome analysis showed the protecting functions of IMB patients against hypoxia-induced oxidative injury in the intestine of Tibetans via affecting GRB2/EGFR/PTPN11 pathways.

## 1. Introduction

Intestinal mucosa is more likely to be damaged if the person is living in an altitude above 3000 meters. Animal experiment showed that high-altitude hypoxia induced impaired intestinal mucosal barrier (IMB) [1, 2]. The incidence of digestive system disease has been reported to be increased in the residents who live at high-altitude environments [3]. Impaired IMB threaten the residents living at high places. The therapy for impaired IMB complicated. The main problem is that the mechanism for high-altitude inducing IMB injury remains widely unknown and no practical therapeutic method can be used yet.

The intestinal tract is an important barrier for preventing bacterial translocation and endotoxin entering human organs. It is well known that intestinal mucosal injury may decrease the function of IMB. The most significant changes in oxygen level in living environments, such as from normobaric normoxia to hypobaric hypoxia (3450 m terrestrial altitude), will result in the increase of reactive oxygen species (ROS) when the balance between prooxidant and antioxidant activity is impaired following exposure to terrestrial hypobaric hypoxia [4]. ROS play an important role in chronic intestinal inflammatory diseases production by increasing the permeability of the endothelium and the mucosa and allowing infiltration of inflammatory leukocytes into intestinal area. Scavenging of ROS is beneficial for intestinal disease [5]. Most Tibetans live at high-altitude plateaus with hypoxic environments [6]. In the environment, many tissues produce ROS, which may arise under the conditions of hypoxia [7]. On the other hand, it has been reported that high-level ROS induces intestinal cell apoptosis [8]. Thus, it almost seems like that ROS levels are aberrant in the intestinal mucosal barrier of Tibetans. ROS has been well known to be associated with oxidative stress [9–12] by damaging lipids, proteins, and DNA [13]. Oxidative stress is an important contributor to the damage of vascular cells [14] and the pathogenesis of hypoxia/reoxygenation injury [15]. Intestinal oxidative stress also is a main factor contributing to intestinal injury, resulting in endotoxin translocation [16]. Dysfunction of the intestinal barrier has been reported to be associated with high-level intestinal oxidative stress [17]. High altitude often induces oxidative stress by affecting biochemical metabolisms, such as lipid metabolism dysfunction [10]. Thus, impaired IMB in the people living at high altitude may be linked to altered control of oxidative stress.

Oxidative stress causes the injury of intestinal tissues via multiple signaling pathways. For example, increased oxidative stress induces the damage in the small intestine of male Sprague-Dawley rats by activating the pathway of p38 mitogen-activated protein kinase [18]. Another example, ischemia/reperfusion results in oxidative injury in animal's intestine. Chinese jujube polysaccharides showed good enzyme activities and ameliorated the injury of the small intestine in rabbits with ischemia/reperfusion [19]. However, most experiments on oxidative stress-induced injury for intestine have been performed in animals and the molecular mechanisms remain unclear for hypobaric hypoxia promotes intestinal barrier dysfunction in the residents at high altitude. Additionally, the molecular mechanisms for hypobaric hypoxia causing the dysfunction of intestinal barrier and development of impaired IMB remain unknown.

The oxidative stress-induced intestinal injury may be associated with many signaling pathways. It is impossible to resolve such complex issues using a single signaling pathway. Transcriptome has been widely used to explore the related pathways in human various diseases [20–22]. To comprehensively understand the effects of hypoxia on IMB in the residents at high altitude, transcriptome experiment was performed here. These gene expression differences were analyzed using DNA microarray. The work will provide important information for hypoxia inducing impaired IMB of the patients at high altitude and basic knowledge for causing IMB injury. The gene expressing profiles of intestinal mucosa from the Tibetans at high altitude more than 3650 m and Han Chinese at 100 m altitude were analyzed to investigate the potential molecules involved in the pathophysiology of IMB injury caused by hypoxic environments. The database for gene ontology (GO) or Kyoto Encyclopedia of Genes and Genomes (KEGG) was referred to predict the genes involving important functions and signaling transduction. Meanwhile, we observed the microstructure of intestine mucosa of Tibetans at high altitude and Han Chinese from plain area with a normal and an electron microscope.

## 2. Material and Methods

### 2.1. Materials

The Trizol reagent was purchased from Life Technologies (Carlsbad, CA, USA). All primers were synthesized by Takara Biotechnology (Dalian) Co., Ltd. (Dalian, China). The cDNA reverse transcription kit and Takara Bio SYBR Premix Ex Taq were from Takara too. Hematoxylin and eosin (H&E) dyes were purchased from Sigma (St. Louis, MO, USA).

### 2.2. Participants

All the protocols in present study were specially approved by Human Research Ethical Committee from the People's Hospital from the Tibet (Tibet, China). All experiments were in compliance with the World Medical Association Declaration of Helsinki regarding ethical conduct of research involving human subjects. From June 2013 and August 2013, 3 Han Chinese from plain area were recruited at Guangzhou First People's Hospital (Guangzhou, China), and 3 Tibetans at high altitude more than 3650 m were recruited at People's Hospital from Tibet (Tibet, China). Each patient had the similar parameter to a healthy participant on gender, birthplace, work intensity, and so on. Research objects were native Tibetans at Lhasa with the age of 40–45. Each participant would sign a consent form before his intestinal mucosa could be taken.

### 2.3. Sample Extraction

The biopsies of mucosa were taken at People's Hospital of the Tibet (Tibet, China) and the Guangzhou First People's Hospital (Guangzhou, China), respectively. Six intestinal biopsies were obtained from the IMB of 10 Tibetans at high altitude more than 3650 m as an experimental group and 10 Han Chinese at 100 m altitude as a control group. All the participants were with underlining normal mucosa. The samples were frozen using liquid nitrogen and kept at −80°C.

### 2.4. The Observation of Intestinal Mucosa by Scanning Electron Microscope

Specimens of sigmoid colon mucosa were obtained at colonoscopy examination from all participants. Twenty samples were used in present work. The samples were fixed with formalin. Ten mm pieces of samples were washed and fixed using osmium tetroxide. All the blocks were made for subsequent histological analysis. Thirty sections were made from one block, dewaxed, dried, and coated using gold palladium by a vacuum evaporator. The microstructure of final samples was observed by a FEI Quanta 400 scanning electron microscope (FEI Company, Hillsboro, OR, USA).

### 2.5. Histological Analysis

All intestinal samples were rinsed with saline solution, fixed in ten percent formaldehyde at 4°C for one day, and washed with PBS. The treated samples were made as four *μ*m species and dyed using H&E stain (hematoxylin and eosin). The microstructure of samples was observed under a microscope. The amounts of villi were calculated within one visual place. IMB was assessed in a double-blind way. The mucosae were injured if intestinal surface was discontinuous, gland was dilated, or superficial cells were damaged [12].

### 2.6. RNA Extraction

Intestinal samples from 10 Tibetans and 10 Han Chinese were collected. All samples were digested in three mL Trizol and ground using a homomixer. Chloroform was added, and RNAs were collected by addition of ethanol. Final RNA samples were resuspended in a buffer with ten mM tris hydrochloride, pH 8.0, one mM EDTA. The quantity of RNA verified by a NanoDrop 1000 Spectrophotometer V3.7 (NanoDrop Technologies, Inc. Wilmington, DE).

### 2.7. RNA Microarray

Genome Oligo Microarray represents the genes and transcripts, which are determined by genome sequencing. RNA microarrays are often regarded as cDNA database after the reverse transcription. All the sequences were obtained from six participants (3 Han Chinese and 3 Tibetans) and verified by aligning these sequences from all known mRNA sequences.

### 2.8. RNA Amplified, Labeled, and Hybridized with Agilent Microarrays

Sample labeling and hybridization was conducted based on the protocols for Microarray-Based Gene Expression. All RNAs were increased and marked by Cy3-UTP. The amount and cRNAs activity were identified using NanoDrop 1000 Spectrophotometer V3.7 (NanoDrop Technologies, Inc., Wilmington, DE). One *μ*g labeled cRNAs were disrupted and then incubated at 60°C within half an hour. Subsequently, cRNA was diluted by GE Hybridization buffer. RNA microarray was assembled by adding 100 *μ*L hybridization solution to the slide. The final sample was heated at 65°C and measured by using Agilent Microarray Scanner.

We used the software for Agilent Feature Extraction to assess all the final data. DEGs (differently expressed genes) were confirmed by a Volcano Plot. Hierarchical Cluster analysis was conducted by the software Agilent GeneSpring GX. Signaling transduction was analyzed and the enrichment in the microarray was calculated.

### 2.9. Enrichment Analysis of Gene Ontology (GO) and Kyoto Encyclopedia of Genes and Genomes (KEGG)

The GO work offers control vocabularies to indicate the DEGs functions. GO has 3 parts: biology processing (BP), cell components (CC), and molecules functions (MF). *P* values showed the richness of DEGs. There were significantly statistical differences if *P* < 0.05. EASE scores, *P* values of Fisher, or hypergeometer presents the significance for the correlated pathways.

### 2.10. Real-Time Quantitative PCR (qRT-PCR)

To further confirm above RNA microarray data, qRT-PCR was used to analyze the top DEGs in 10 Han Chinese at 100 m altitude and 10 Tibetans at high altitude more than 3480 m, including epidermal growth factor receptor (EGFR), growth factor receptor-bound protein 2 (GRB2), and tyrosine-protein phosphatase nonreceptor type 11 (PTPN11). The RNAs were extracted using above intestinal mucosa by Trizol. Five *μ*g RNA was reversely transcribed using reverse transcription kits. All the primers were given as Table 2 showed, and qRT-PCR was conducted by SYBR® Green RT-PCR Kit on the real-time PCR system. The amplification situation was given as follows: 94°C for 5 min, 45 cycles of 95°C for 20 s, 65°C for 30 s, and 65°C for 40 s.

### 2.11. Western Blot Analysis

Rabbit anti-human polyclonal EGFR antibody (Cat. number ab2430), rabbit anti-human polyclonal GRB2 antibody (ab32037), rabbit anti-human monoclonal PTPN11 antibody (Cat. number ab32083), and rabbit anti-human beta-actin antibody (Cat. number ab8227) and goat anti-rabbit HRP (IgG H&L) (Cat. number ab6721) were purchased from Abcam Shanghai office launch (Shanghai, China). The intestinal samples were taken from all participants by a noninvasive method using endoscopic techniques. All tissues were ground by using a sterile mortar and pestle. Sample proteins were collected by centrifugation and separated by SDS-PAGE and then electrophoretically transferred onto PVNF membranes. After blocking the membranes with free-fat milk, they were then incubated with primary antibody. The members were washed three times and incubated with HRP-linked secondary antibody. The protein expression level was normalized by beta-actin expression. The immunoreactive result was visualized by using an enhanced chemiluminescence system (Amersham Pharmacia Biotech, Stockholm, Sweden).

### 2.12. The Locations of DEGs on Human Chromosomes

The locations of DEGs on human chromosomes were marked on the human chromosomes using the data from RNA microarray results. Messenger RNA expression profiles were analyzed at genome level using the above results. About 400 significantly differently expressed genes were marked on 24 human chromosomes. The fold changes were marked with different colors.

### 2.13. Data Analysis

All data were showed using average values ± S.D. An ANOVA analysis was performed to compare the difference between different groups and the statistical significance was verified. There were significantly statistical differences if *P* < 0.05.

### 2.14. Construction of Gene Networks Based on Microarray Data

The significantly differentially expressed genes from microarray data were used. String software was used to retrieve the interacting genes (http://string-db.org/). Up- or downregulated genes from the microarray were visualized on this network. According to experimental results and computational prediction, a confidence score was used to confirm the interaction between miRNA and DEGs. The confidence score > 0.5 is regarded as statistically significant.

## 3. Results

### 3.1. Baseline Characteristics of Participants

The baseline and physical characteristics of the study population were listed in Table 1. There were significant differences for the distribution of living altitude (*P* < 0.01) but no difference for years at their locations (*P* > 0.05) between Tibetans and Han Chinese. In contrast, no significant difference was found for other parameters including age, cigarette smoking, alcohol drinking, and BMI (*P* > 0.05, Table 1). In contrast, there were statistically significant differences for Hb and diastolic pressures (*P* < 0.05, Table 1). The levels of oxygen saturation were lower in the residents from high altitude than at low altitude (*P* < 0.01), although heart rates in Tibetans were faster than in Han Chinese.

### 3.2. Hematoxylin-Eosin Staining Analysis of Intestinal Tissues

**Figure 1:**
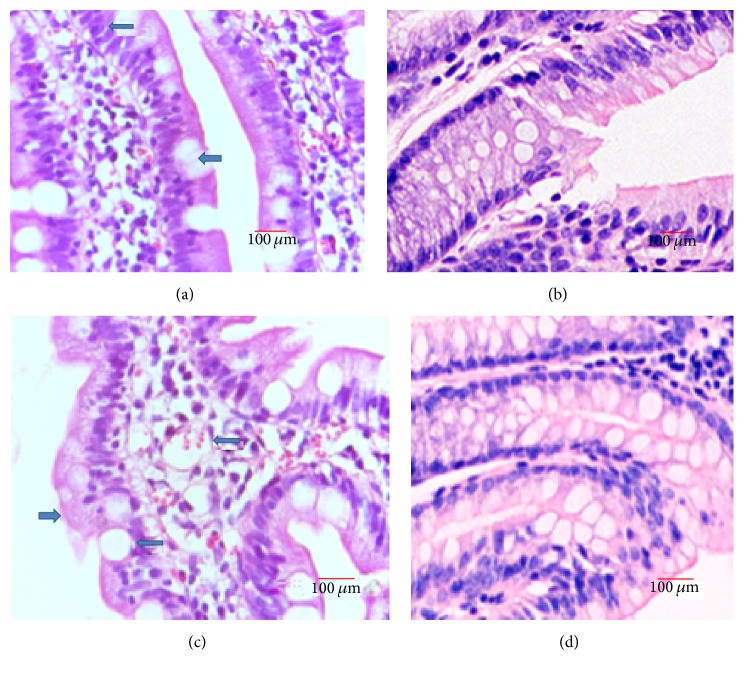
Histopathological examination of intestinal mucosa sections. (a) Cylindric and cup cells were mostly destroyed as arrow indicated in the IMB of Tibetans. (b) Cylindric and cup cells had normal structures in the IMB of Han Chinese. (c) There were more capillary microvessels as arrow indicated in the intestinal mucosa in antrum region of the Tibetans. (d) There were no more capillary microvessels as arrow indicated in the intestinal mucosa in antrum region of Han Chinese.

Hematoxylin-eosin stained results showed cylindric and cup cells were mostly destroyed as arrow indicated in Tibetans (Figure 1(a)) while cylindric and cup cells had normal structures in Han Chinese (Figure 1(b)). There were more capillary microvessels in the intestinal mucosa in antrum region of the Tibetans than in Han Chinese (Figure 1(c)) while there were no more capillary microvessels in the intestinal mucosa in antrum region of Han Chinese (Figure 1(d)).

### 3.3. Scanning Electron Microscope (SEM) Analysis of Intestinal Mucosa

**Figure 2:**
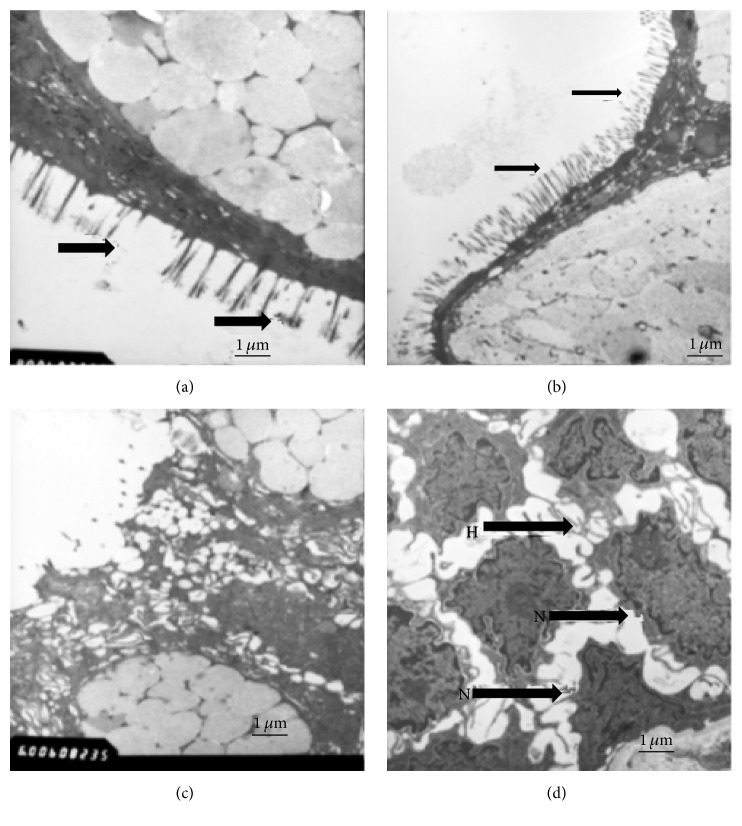
Electroscopic studies of the digestive tract in the IMB of Tibetans and Han Chinese at high altitude. (a) Intestinal villi are usually reduced and appear irregular in the IMB of Tibetans. (b) Intestinal villi are usually rich in the Han Chinese. (c) Glandular epithelium is destroyed in the IMB of Tibetans. (d) Glandular epithelium is in a fine situation in Han Chinese.

SEM of the intestinal mucosa from jejunum was shown in Figure 2, revealing the basic characteristics of the intestinal tissues. The features of intestinal symptoms were seen in these specimens. Electromicroscopy showed that Tibetans had intestinal mucosa injury while Han Chinese had normal intestinal mucosa. Intestinal villi were usually reduced and appeared irregular in the IMB of Tibetans (Figure 2(a)) while intestinal villi were usually rich and in regular form in the IMB of Han Chinese (Figure 2(b)). Glandular epithelium was destroyed in the IMB of Tibetans (Figure 2(c)) while the glandular epithelium was in a fine situation in the IMB of Han Chinese (Figure 2(d)).

### 3.4. Screening of Differentially Expressed Genes

**Figure 3:**
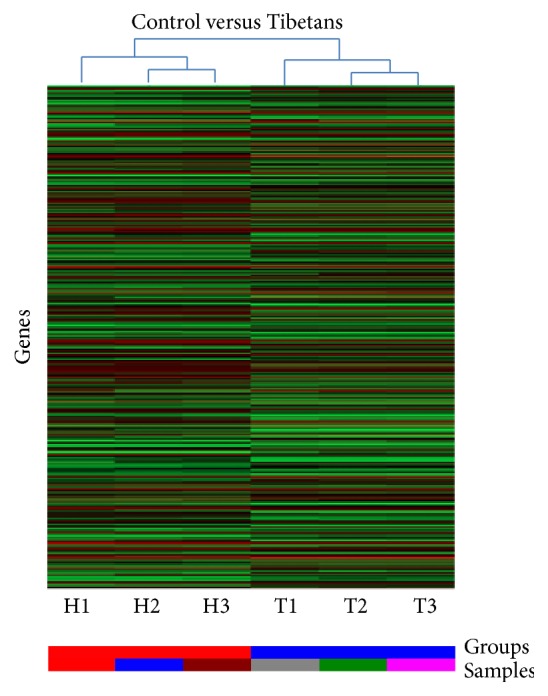
Hierarchical cluster analysis of the altered genes in the intestinal mucosa of IMB of Tibetans and Han Chinese. The color code in each heat map has been lineared with green as the lowest level for mRNA and red as the highest level for mRNA. The increased genes expression was shown in green to red, whereas the decreased genes expression was shown from red to green.

Hierarchical cluster analysis was conducted as Figure 3 showed. Three Han Chinese and three high-altitude Tibetans showed different gene expressing patterns. The levels of 1237 genes increased and 1336 genes decreased in the intestinal tissues from Tibetans when compared with Han Chinese. There were 25-, 22-, and 18-fold downregulation for GRB2, EGFR, and PTPN11 in the IMB from Tibetans when compared with Han Chinese.

### 3.5. qRT-PCR

**Figure 4:**
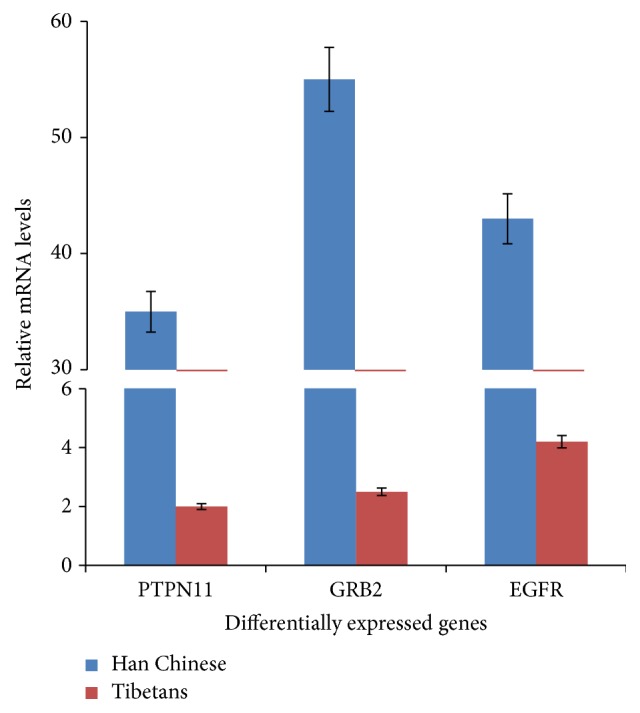
Validation of microarray results (the top 3 up- and downregulated DEGs) by qRT-PCR. The results represented quantification of mRNA levels relative to beta-actin. Normalized expression values were obtained by qRT-PCR (*n* = 10). *C* = Han Chinese at 100 m altitude and **P** = Tibetans at high altitude more than 3480 m. All the data were present as average value ± SD. *P* < 0.05 via IMB of Tibetans.

We measured the levels of three significantly changed genes using real-time PCR. GRB2, EGFR, and PTPN11 had similar expressing profiles with those obtained from the RNA transcriptomes analysis. GRB2, EGFR, and PTPN11 were more than 20-fold downregulated in Tibetans when comparing to Han Chinese (*P* < 0.05) (Figure 4). The results showed the similar changing trend with that from microarray analysis while their baseline characters were also similar with patients analyzed by microarray method (Table 1).

### 3.6. Protein Expression of GRB2, EGFR, and PTPN11

**Figure 5:**
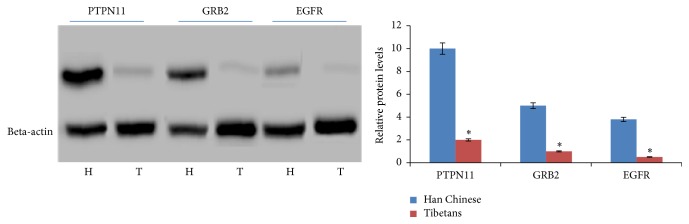
Validation of microarray (the top 3 up- and downregulated DEGs) results by Western Blot. The results represented quantification of protein levels relative to beta-actin. Normalized expression values were obtained by Western Blot (*n* = 10). *C* = Han Chinese at 100 m altitude and **P** = Tibetans at high altitude more than 3480 m. All the data were present as average value ± SD. ^*∗*^ *P* < 0.05 via IMB of Tibetans.

The protein levels of GRB2, EGFR, and PTPN11 had similar changing trends with those obtained from qRT-PCR analysis. GRB2, EGFR, and PTPN11 were significantly downregulated in Tibetans when comparing to Han Chinese (*P* < 0.05) (Figure 5). The results showed the similar changing trend with those from microarray analysis.

### 3.7. Comprehensive Peptidome Profiling of DEGs

**Figure 6:**
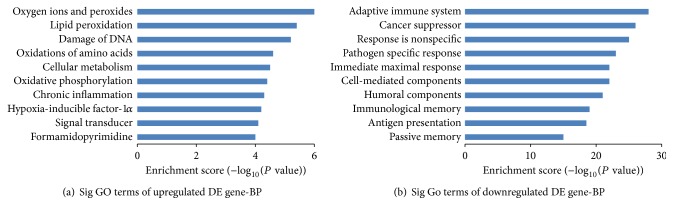
Gene ontology (GO) analysis used for analysis of the altered genes. (a) The bar plot shows the top ten upregulated Enrichment Score values of the significant enrichment. (b) The bar plot shows the top ten downregulated Enrichment Score values of the significant enrichment BP.

**Figure 7:**
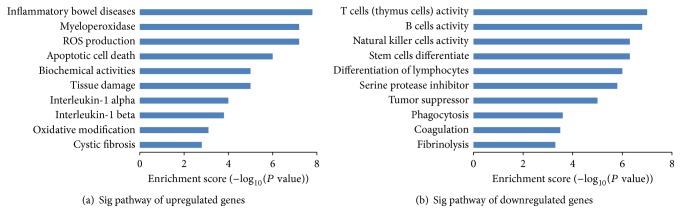
Pathway analysis of DEG. (a) The bar plot shows the top ten upregulated Enrichment Score values of the significant enrichment pathway. (b) The bar plot shows the top ten downregulated Enrichment Score value of the significant enrichment pathway.

All DEGs were explained using GO terms by peptidome profiling analysis. All the events were elucidated in intestinal mucosa from the IMB of Tibetans when compared to those from Han Chinese. In the GO, the upregulated DE genes were involved in the reactive oxygen species activity, oxygen ions and peroxides, lipid peroxidation and so on; the downregulated DE genes were involved in the decrease of immunological ability. All the changes can be caused by oxidative stress, such as cancer suppressor [23], cell normal functions [24], special responses for pathogens [25], maximal actions, and antigen presentation [26] (Figure 6). Pathway analysis revealed that, in intestinal mucosa tissues of participants from high altitude, many pathway genes had aberrant expression and may be also related with oxidative stress, especially in inflammatory bowel diseases [27, 28], myeloperoxidase [29], ROS production [10], apoptotic cell death [30], tissue damage [31], interleukin-1 [32], oxidative modification [33], cystic fibrosis [34], natural killer cells activity [35], lymphocytes [36], fibrinolysis [37] and so on (Figure 7).

### 3.8. Clusters of Differently Expressed Genes on Human Chromosomes

**Figure 8:**
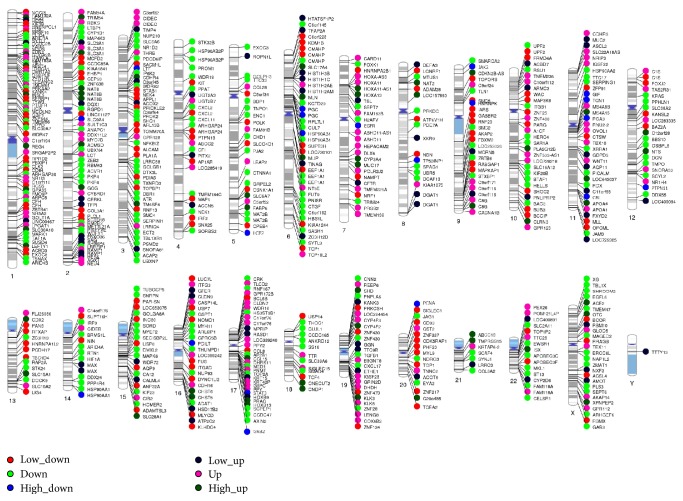
Whole-chromosome bird-view of expression levels of the 400 top DEGs located to 23 chromosomes. The color of each circle stands for the relative level of one DEG. Expression levels are normalized to six grades (low-up/4–6-fold, up/7–10-fold, and high up/more than 10-fold; low-down/4–6-fold, down/7–10-fold, and high down/more than 10-fold).

Just as Figure 8 showed, four hundred DEGs are mapped on using human chromosomes. The differently expressed genes on chromosomes thirteen, fourteen, fifteen, eighteen, twenty, twenty-one, twenty-two, and Y had fewer DEGs locations involved with reactive oxygen species activities. The high density of DEGs gathered on chromosomes one, six, seven, eleven, fourteen, seventeen, and nineteen. The most significantly expressed genes with more than 10-fold were all located on three different chromosomes: epidermal growth factor receptor (EGFR), chromosome 7p12.3-p12.1; growth factor receptor-bound protein 2 (GRB2), chromosome 17q24-q25; and tyrosine-protein phosphatase nonreceptor type 11 (PTPN11), chromosome 12q22-qter (Figure 8).

### 3.9. Visualization of Microarray Data by Using DEGs Networks

**Figure 9:**
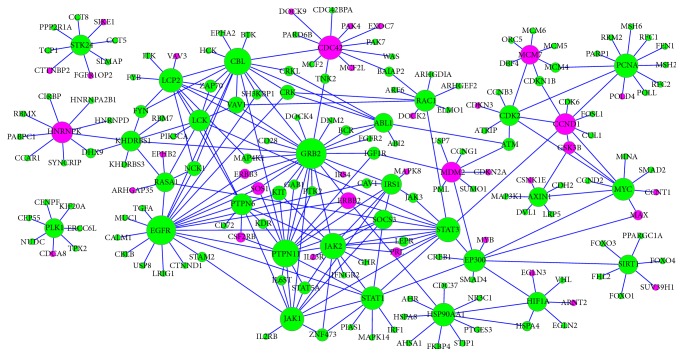
Gene network of top downregulated and upregulated DEGs from PCR microarray data. Significantly regulated genes were shown as purple and green, respectively. The size of circle represented the expression level.

The microarray data mainly showed the up- and downregulated genes and were visualized on the network (Figure 9), which was created around mainly interesting proteins relating to the protecting functions for oxidative stresses and significantly differently expressed between Tibetans and Han Chinese. On the network, genes that were downregulated were shown as green circles and genes that upregulated were shown as red circles (Figure 9). In Tibetans, three main signaling pathways associated with GRB2, EGFR, and PTPN11 were shown on a network to be significantly downregulated when compared with Han Chinese (Figure 9).

## 4. Discussion

Many factors can result in the injury of intestinal mucosa and hypoxia is an important risk for causing the injury of intestinal tissues [1, 38–42]. Firstly, hypoxic pressure affects basic metabolic processes [43], resulting in the changes for many biological functions. Secondly, hypoxia-caused carbonic anhydrase, which constitutes an acidic microenvironment [44], is harmful to the residents at high altitude. Thirdly, hypoxia environment disturbs the gut flora imbalance in the residents at high altitude causing the intestinal injury [45]. Hypobaric hypoxia will inhibit the secretion of IgG, which is an important immune-related molecule in intestine [46, 47]. Furthermore, hypobaric hypoxia also inhibits bile secretion and decreases enterohepatic circulation, resulting in intestinal dysfunction and bacterial overproliferation and increasing the damage of intestinal biological barriers [48]. Especially for the first point, hypoxic pressure affects basic metabolic processes and produces high-level ROS, which lead to the increase of oxidative stress [49, 50]. Oxidative stress is an important contributor for tissue injuries, including intestine injury [18, 51].

*Homo sapiens* Genome Oligo Microarray has most well-known genes for human being. All data can be compared with the resourceful sequences with clear functions [52–54]. We use the data to explore the expressing profiles of intestinal tissues from Tibetans at high altitude. Meanwhile, the data compared with those from Han Chinese at plain area were analyzed using genome microarrays, in which 1137 DEGs increased while 1436 DEGs decreased with more than two-fold changes in the Tibetans. Based on those data, we then studied differentially expressed genes function. Our results indicated that IMB involves the oxidant responses.

Present findings indicated that GRB2/EGFR/PTPN11-associated pathways were significantly downregulated (fold change = 25, 22, and 18). As reported in previous studies, GRB2 was related with formation of reactive and oxidative products [55, 56]. ROS are directly involved in gastrointestinal injury. High concentration of ROS in intestinal mucosa possibly decreases mucosal organ-protective efficacy. Many factors, such as intestinal food, are more likely to destroy the mucosal structure when intestinal mucosa is in a fragile condition [57]. Meanwhile, ROS increase the permeability of small intestinal epithelial cells and lead to intestinal mucosal injury at an early stage [58].

The inhibition of GRB2 can significantly reduce fat accumulation, improve glucose metabolism, ameliorate oxidative stress [55], and activate mitogen-activated protein kinase pathways [59]. Additionally, GRB2 deficiency reduces cell apoptosis by inactivating caspase-3. The decrease in GRB2 improves hepatic steatosis and glucose metabolism and reduces oxidative stress. All these activities will improve the intestinal injury induced by hypoxia-induced oxidative stress.

Despite the important role of EGFR in intestinal epithelial cells [60], the study on the effects of EGFR on the intestinal injury is very limited. According to a previous report, the overexpression of EGFR increases the levels of ROS, accumulates DNA strand damage, and makes genome unstable. The levels of EGFR activation are associated with oxidative stress [61]. Therefore, inactivation of EGFR pathway will decrease the level of ROS and reduce the oxidative stress. The downregulation EGFR pathway will improve the protecting functions for intestinal injury.

The role of PTPN11 pathway is seldom reported in intestinal tissues. From the network, PTPN11 is closely associated with the JAK and STAT signaling pathways (Figure 9), which are activated by protein tyrosine kinases and phosphatases, and is necessary in regulating cellular activities responding various cytokines. Dysregulation of the JAK and STAT pathways will lead to hematopoietic and immune diseases. PTPN11 plays an important regulatory role in JAK and STAT signaling pathways [62]. The JAK2 and STAT pathways have been reported in cell protection and injury. The JAK2 inhibitor and overexpression of its dominant negative JAK2 protein improve endothelial cells against peroxide and superoxide anion. Inactivation of JAK2 has been proved to be a potential method for endothelial cells against oxidative stress-induced death [63]. Parthenolide has been reported to inhibit JAK1 and STAT3 activity. ROS product will inhibit STAT3 signaling pathway by targeting JAK1 [64]. From the network, PTPN11 can regulate JAK and STAT pathways, and its inhibition will contribute to prevent oxidative-induced injury for the intestine of Tibetans. On the other hand, there is also different report for PTPN11. Gain of function mutations of PTPN11 in hematopoietic cells caused cytokine hypersensitivity by enhancing the levels of ROS. PTPN11 mutations will improve mitochondrial aerobic metabolism via the interaction with a new molecule. The mutation of PTPN11 has a therapeutic benefit by improving antioxidant activities [65].

One question should be paid here. There were 1336 downregulated genes but only three downregulated genes GRB2/EGFR/PTPN11 were selected. Three top-changed genes were analyzed because all of them were more than 20-fold downregulated while the left is less than 10-fold downregulated. Furthermore, the three genes were closed associated with the ROS production. The generation of ROS is tightly regulated by GRB2 in colorectal tumorigenesis [56]. ROS production will be beneficial to EGFR activation [66]. A conditionally deleted allele of PTPN11 will result in lower ROS levels [67]. Thus, the three genes were analyzed in the work.

From above results, it is easy to find that the three pathways have similar functions for controlling ROS levels and inhibiting oxidative-induced injury for human tissues or cells. Furthermore, our network also shows the close relationship among GRB2, EGFR, and PTPN11 pathways (Figure 9), which is accordant with previous reports [68, 69] except of PTPN11 pathway. Further work is needed to confirm the detailed relation among the three pathways.

There are some limitations for present study: (1) we only recruited a few participants from each group (living at altitude versus not). This is not remotely representative of the larger human population living within this region. To avoid the values bias caused by small sample size, the results were confirmed by using qRT-PCR in 10 Han Chinese at 100 m altitude and 10 Tibetans at high altitude more than 3480 m and were stable when compared with those of microarray analysis (Figure 4). (2) Present results only reflect one aspect for the differences noted in the study. There are still other molecular mechanisms existed, such as phenotype differences between Tibetans and Han Chinese or fundamentally different lifestyles. It would have been more relevant to study the same individuals moving from the plains into Tibet and vice versa. To address this issue, we tried such work for many times and always failed finally. The main reason was caused by the fact that most persons from plain cannot stay longer at high-altitude places. Furthermore, to reduce the disturbance, the lifestyle (similar daily activity, food calorie intake, and so on) and occupation (office workers) are similar between groups. Actually, Tibetan and Han Chinese populations diverged less than 3,000 years [70], suggesting that most genes are stable.

## 5. Conclusions

Present findings are obtained by comparing the gene expressing profiles of the participants from high altitude and plain area, providing clues to the molecular pathogenesis of this condition. Genome-wide transcriptional analysis suggests that hypoxia-induced oxidative stress leads to the intestinal injury of Tibetans via the inhibition of GRB2/EGFR/PTPN11 pathways. The study provides important information for the molecular mechanism causing IMB injury at high altitude and lays a foundation for subsequent gene validation and functional researches.

## Figures and Tables

**Table 1:** The baseline characters of all participants.

Characteristic	Han Chinese		Tibetans		*F*-ratio	*P* value
	Group I	Group II	Group I	Group II		
Age (years)	41–45	40–49	40–45	39–48	0.38	0.77
Smoking (no/yes)	1/2	3/4	1/2	2/5	1.77	0.29
Drinking (no/yes)	1/2	3/4	1/2	2/5	1.77	0.29
Gender (male/female)	2/1	5/2	2/1	4/3	1.77	0.29
BMI	29–34	28–35	30–33	27–36	0.26	0.29
Food calorie intake (kcal/d)	2545.4–2089.3	2435.6–2132.7	2533.8–2134.7	2510.6–2184.1	0.85	0.36
Frequency of food (per day)	3 times	3 times	3 times	3 times	0	1
Habit	Rural	Rural	Rural	Rural	—	—
Marital status	Married	Married	Married	Married	—	—
Physical activity	Routine work	Routine work	Routine work	Routine work	—	—
Emotional makeup	Normal	Normal	Normal	Normal	—	—
Sleep habits	Day	Day	Day	Day	—	—
Mental stress	Social	Social	Social	Social	—	—
Water intake	During meal	During meal	During meal	During meal	—	—
Diet and sleep timings	Regular	Regular	Regular	Regular	—	—
Living at altitude (meters)	100	100	3650–3690	3650–3690	297.3160	<0.0001
Time at plain area or high altitude (years)	41–45	40–46	40–45	42–44	1.54	0.28
Hb (g/L)	144–156	140–160	166–175	165–180	2.56	0.04
Systolic pressure (mmHg)	112–124	108–129	125–130	122–138	2.20	0.03
Diastolic pressure (mmHg)	68–78	65–80	82–89	80–92	3.62	0.01
Blood oxygen saturation (%)	98-99	98-99	82–85	80–86	5.36	0.01
Heart rate (time/mini)	64–78	60–80	78–87	78–90	2.04	0.06

Note: BMI, body mass index; Hb, hemoglobin. There is a significant difference if *P* < 0.05.

Group I, the participants underwent genome-wide transcriptional analysis, real-time PCR, and Western Blot analysis. Group II, the participants underwent real-time PCR and Western Blot analysis.

**Table 2:** The primers used for real-time quantitative PCR.

Genes	GenBank accession number		Primers (5′-3′)	Size (bp)
EGFR	BC094761.1	Forward	accatccaggaggtggctgg	440
		Reverse	ggatcacacttttgtccctg	

GBR2	JX512444.1	Forward	aagacggcttcattcccaag	134
		Reverse	ctctctcggataagaaaggc	

PTPN11	NM_002834.3	Forward	ttcacactttccgttagaag	162
		Reverse	attgcccgtgatgttccatg	

Note: epidermal growth factor receptor (EGFR), growth factor receptor-bound protein 2 (GRB2), and tyrosine-protein phosphatase nonreceptor type 11 (PTPN11).
